# Supplementary material for: Hot spot prediction in protein-protein interactions by an ensemble system
Source: BMC Syst Biol. 2018 Dec 31;12(Suppl 9):132. doi: 10.1186/s12918-018-0665-8 (PMC6311905; doi:10.1186/s12918-018-0665-8)
Supplement: Supplementary file 2 — All relevant MATLAB code. A simple Matlab implement of our predictor. (ZIP 19,742 kb) [file 12918_2018_665_MOESM2_ESM.zip › Additional file 2/Read ASA/ASA website/ASEdb/1NMBL.htm]

 NetSurfP - Protein Surface Accessibility and Secondary Structure Predictions

  

|  |  |  |
| --- | --- | --- |
|  |  | NetSurfP - Protein Surface Accessibility and Secondary Structure Predictions  Technical University of Denmark |

  


---

```
# For publication of results, please cite:
# A generic method for assignment of reliability scores applied to solvent accessibility predictions.
# Bent Petersen, Thomas Nordahl Petersen, Pernille Andersen, Morten Nielsen and Claus Lundegaard
# BMC Structural Biology 2009, 9:51 doi:10.1186/1472-6807-9-51
#
# Column 1: Class assignment - B for buried or E for Exposed - Threshold: 25% exposure, but not based on RSA
# Column 2: Amino acid
# Column 3: Sequence name
# Column 4: Amino acid number
# Column 5: Relative Surface Accessibility - RSA
# Column 6: Absolute Surface Accessibility
# Column 7: Z-fit score for RSA prediction
# Column 8: Probability for Alpha-Helix
# Column 9: Probability for Beta-strand
# Column 10: Probability for Coil
E D  1NMBL                  1    0.821 118.321   0.705   0.003   0.003   0.994
B I  1NMBL                  2    0.269  49.784  -0.808   0.004   0.514   0.481
E Q  1NMBL                  3    0.469  83.781   0.835   0.002   0.816   0.182
B M  1NMBL                  4    0.153  30.695   0.445   0.002   0.816   0.182
E T  1NMBL                  5    0.448  62.193   0.325   0.003   0.718   0.279
B Q  1NMBL                  6    0.140  25.040   0.169   0.004   0.420   0.576
E T  1NMBL                  7    0.427  59.267   0.157   0.004   0.085   0.910
E T  1NMBL                  8    0.499  69.170  -1.058   0.018   0.019   0.964
E S  1NMBL                  9    0.526  61.635  -1.273   0.053   0.043   0.903
E S  1NMBL                 10    0.365  42.731  -1.171   0.022   0.359   0.619
B L  1NMBL                 11    0.206  37.664  -0.078   0.021   0.756   0.223
E S  1NMBL                 12    0.404  47.396  -0.654   0.021   0.756   0.223
B A  1NMBL                 13    0.248  27.363  -0.062   0.023   0.655   0.322
E S  1NMBL                 14    0.412  48.275   0.364   0.005   0.336   0.660
E L  1NMBL                 15    0.505  92.484   0.205   0.004   0.085   0.910
E G  1NMBL                 16    0.603  47.448  -1.342   0.005   0.045   0.951
E D  1NMBL                 17    0.428  61.732   0.317   0.004   0.197   0.799
E R  1NMBL                 18    0.536 122.813   1.019   0.003   0.718   0.279
B V  1NMBL                 19    0.081  12.419   0.364   0.001   0.900   0.099
E T  1NMBL                 20    0.403  55.854   1.115   0.001   0.959   0.040
B I  1NMBL                 21    0.048   8.806   0.171   0.001   0.959   0.040
E S  1NMBL                 22    0.340  39.848   0.540   0.001   0.900   0.099
B C  1NMBL                 23    0.027   3.819   0.200   0.001   0.900   0.099
E R  1NMBL                 24    0.394  90.249   0.902   0.002   0.816   0.182
B A  1NMBL                 25    0.238  26.228  -1.415   0.002   0.816   0.182
B S  1NMBL                 26    0.256  30.038  -1.368   0.004   0.616   0.381
E Q  1NMBL                 27    0.475  84.764  -1.866   0.004   0.138   0.858
E D  1NMBL                 28    0.434  62.496  -2.550   0.018   0.047   0.935
E I  1NMBL                 29    0.451  83.454  -2.007   0.018   0.088   0.893
E S  1NMBL                 30    0.385  45.075  -1.571   0.018   0.088   0.893
B N  1NMBL                 31    0.246  35.970  -0.712   0.005   0.262   0.733
B Y  1NMBL                 32    0.105  22.503   0.480   0.004   0.616   0.381
B L  1NMBL                 33    0.027   5.035   0.460   0.002   0.816   0.182
B N  1NMBL                 34    0.067   9.780   0.387   0.011   0.918   0.071
B W  1NMBL                 35    0.031   7.480   0.291   0.011   0.918   0.071
B Y  1NMBL                 36    0.039   8.377   0.639   0.011   0.918   0.071
B Q  1NMBL                 37    0.141  25.254   0.843   0.021   0.756   0.223
B Q  1NMBL                 38    0.078  13.895   0.315   0.004   0.514   0.481
E N  1NMBL                 39    0.402  58.838   1.761   0.005   0.045   0.951
E P  1NMBL                 40    0.324  45.976   0.002   0.005   0.015   0.979
E D  1NMBL                 41    0.766 110.366  -1.663   0.003   0.003   0.994
E G  1NMBL                 42    0.501  39.437   0.057   0.018   0.019   0.964
E T  1NMBL                 43    0.373  51.763  -0.238   0.018   0.047   0.935
B V  1NMBL                 44    0.098  15.001   0.519   0.020   0.205   0.775
E K  1NMBL                 45    0.289  59.530   0.673   0.021   0.756   0.223
B L  1NMBL                 46    0.097  17.761   0.603   0.011   0.918   0.071
B L  1NMBL                 47    0.022   3.992   1.089   0.011   0.918   0.071
B I  1NMBL                 48    0.022   4.051   0.545   0.001   0.959   0.040
B Y  1NMBL                 49    0.092  19.639   0.144   0.001   0.959   0.040
B Y  1NMBL                 50    0.103  22.011  -0.353   0.001   0.900   0.099
B T  1NMBL                 51    0.178  24.675  -0.803   0.004   0.514   0.481
B S  1NMBL                 52    0.234  27.437  -1.224   0.005   0.262   0.733
E N  1NMBL                 53    0.619  90.666  -1.540   0.004   0.085   0.910
E L  1NMBL                 54    0.434  79.392  -0.600   0.018   0.047   0.935
E H  1NMBL                 55    0.634 115.361  -1.953   0.018   0.019   0.964
E S  1NMBL                 56    0.685  80.294  -1.493   0.018   0.047   0.935
E E  1NMBL                 57    0.586 102.304  -1.490   0.018   0.047   0.935
E V  1NMBL                 58    0.288  44.219  -0.161   0.004   0.085   0.910
E P  1NMBL                 59    0.528  74.881  -1.157   0.004   0.138   0.858
E S  1NMBL                 60    0.330  38.653  -2.282   0.005   0.262   0.733
B R  1NMBL                 61    0.237  54.273  -0.820   0.004   0.616   0.381
B F  1NMBL                 62    0.054  10.778  -0.882   0.002   0.816   0.182
B S  1NMBL                 63    0.172  20.135  -1.242   0.001   0.900   0.099
B G  1NMBL                 64    0.141  11.112  -2.796   0.001   0.900   0.099
E S  1NMBL                 65    0.394  46.200  -0.248   0.003   0.718   0.279
E G  1NMBL                 66    0.431  33.920  -0.866   0.004   0.420   0.576
E S  1NMBL                 67    0.602  70.508  -1.545   0.004   0.138   0.858
E G  1NMBL                 68    0.654  51.486  -0.859   0.005   0.045   0.951
E T  1NMBL                 69    0.420  58.282  -0.195   0.005   0.262   0.733
E D  1NMBL                 70    0.409  58.995   0.664   0.002   0.816   0.182
B Y  1NMBL                 71    0.065  13.955  -0.008   0.001   0.900   0.099
B S  1NMBL                 72    0.250  29.347   0.834   0.001   0.900   0.099
B L  1NMBL                 73    0.030   5.420   0.430   0.001   0.959   0.040
E T  1NMBL                 74    0.312  43.219   1.025   0.001   0.900   0.099
B I  1NMBL                 75    0.076  14.115   0.118   0.002   0.816   0.182
E S  1NMBL                 76    0.454  53.150   0.331   0.004   0.514   0.481
E N  1NMBL                 77    0.543  79.437  -0.535   0.004   0.197   0.799
B L  1NMBL                 78    0.139  25.396   0.167   0.004   0.085   0.910
E E  1NMBL                 79    0.462  80.624   0.609   0.018   0.088   0.893
E Q  1NMBL                 80    0.434  77.477  -0.694   0.018   0.088   0.893
E E  1NMBL                 81    0.530  92.608  -0.878   0.053   0.043   0.903
B D  1NMBL                 82    0.219  31.529  -0.688   0.052   0.084   0.864
B I  1NMBL                 83    0.220  40.644   0.086   0.066   0.296   0.638
B A  1NMBL                 84    0.056   6.215  -0.271   0.021   0.756   0.223
B T  1NMBL                 85    0.181  25.077   0.920   0.018   0.846   0.136
B Y  1NMBL                 86    0.043   9.275   0.941   0.018   0.846   0.136
B F  1NMBL                 87    0.056  11.299   1.164   0.021   0.756   0.223
B C  1NMBL                 88    0.018   2.583   1.217   0.023   0.655   0.322
B Q  1NMBL                 89    0.046   8.233  -0.179   0.023   0.655   0.322
B Q  1NMBL                 90    0.119  21.271  -0.190   0.022   0.552   0.426
B D  1NMBL                 91    0.236  33.964  -0.540   0.021   0.279   0.699
E F  1NMBL                 92    0.504 101.213  -1.698   0.018   0.088   0.893
E T  1NMBL                 93    0.525  72.762  -2.395   0.005   0.045   0.951
E L  1NMBL                 94    0.371  67.893  -1.088   0.018   0.047   0.935
E P  1NMBL                 95    0.414  58.761   0.025   0.020   0.205   0.775
B F  1NMBL                 96    0.203  40.762   0.195   0.022   0.552   0.426
B T  1NMBL                 97    0.143  19.890  -1.221   0.023   0.655   0.322
B F  1NMBL                 98    0.106  21.354  -0.785   0.004   0.616   0.381
B G  1NMBL                 99    0.113   8.932  -2.355   0.005   0.262   0.733
E G  1NMBL                100    0.441  34.730  -0.353   0.004   0.085   0.910
B G  1NMBL                101    0.279  21.989  -1.641   0.004   0.197   0.799
B T  1NMBL                102    0.147  20.431  -0.060   0.004   0.514   0.481
E K  1NMBL                103    0.431  88.739   1.098   0.001   0.900   0.099
B L  1NMBL                104    0.124  22.631   0.700   0.001   0.959   0.040
E E  1NMBL                105    0.338  58.961   0.894   0.001   0.959   0.040
B I  1NMBL                106    0.138  25.549  -0.008   0.001   0.900   0.099
E R  1NMBL                107    0.510 116.721   0.683   0.001   0.900   0.099
E R  1NMBL                108    0.535 122.469  -0.352   0.004   0.514   0.481
E A  1NMBL                109    0.734  80.876  -1.761   0.006   0.001   0.993

Explain the output.  Go back.
```

---
